# Management of latent TB infection: a national survey of French physicians

**DOI:** 10.5588/ijtldopen.25.0368

**Published:** 2025-10-10

**Authors:** N. Dournon, Y. Tandjaoui-Lambiotte, C.M. Kilu, P.T. Phan, P. Fraisse, F.-X. Blanc, O. Launay, O. Epaulard, C. Andrejak, P. Loubet, A. Dinh, Groupe Prévention Vaccination (SPILF) and Groupe de Recherche et d’Enseignement en Pneumo-Infectiologie (GREPI, SPLF)

**Affiliations:** ^1^ Infectious Disease Department, University Hospital, Garches, France;; ^2^ Service de Pneumologie and Infectiologie, CH Saint Denis, INSERM UMR 1137 IAME, Paris, France;; ^3^ INSERM UMR 1272 Hypoxie and Poumon, Bobigny, France;; ^4^ Respiratory Department, Hue National General Hospital, Hue, Vietnam;; ^5^ Pulmonology Department, University Hospital, Strasbourg, France;; ^6^ French National Network of Tuberculosis Centers, Paris, France;; ^7^ L’institut du thorax, Nantes Université, CHU Nantes, Nantes, France;; ^8^ GREPI, SPLF, Paris, France;; ^9^ International Vaccination Center, University Hospital Cochin, APHP, Paris, France;; ^10^ Infectious Disease, University Hospital La Tronche, Grenoble, France;; ^11^ Pulmonology Department, University Hospital, Amiens, France;; ^12^ Virulence Bactérienne et Infections Chroniques, INSERM U1047, University Montpellier Department of Infectious and Tropical Disease, CHU Nîmes, Nîmes, France.

**Keywords:** tuberculosis, TBI, diagnosis, HIV

Dear Editor,

TB remains a major global public health issue.^1,2^ In France, national guidelines for the screening and management of latent TB infection (LTBI) have been updated in 2019.^3^ However, concerns remain regarding their implementation in routine clinical practice.^4^ We conducted a nationwide survey to evaluate how French physicians, specifically infectious disease specialists (IDS) and pulmonologists, declare to diagnose and manage LTBI and to identify barriers to implementation. At last, we are aware that the current terminology in 2025 has shifted towards ‘*Mycobacterium tuberculosis* infection’ rather than LTBI, but in the context of our survey it was the terminology used at the time.^5^

A web-based, self-administered questionnaire was distributed between 1 January 2024 and 1 January 2025, via the French Infectious Disease Society (SPILF), the French-speaking Respiratory Medicine Society (SPLF), and social media platforms.

Developed by a multidisciplinary working group composed of IDS and pulmonologists from national academic centres, the questionnaire aimed to assess current practices and attitudes towards both diagnosis and management of LTBI in France. The final version consisted of 30 closed-ended and multiple-choice questions structured into three main sections: 1) respondent demographics and professional background, 2) diagnostic approaches and preferred screening tools for LTBI, and 3) management strategies across eight distinct clinical scenarios, including health care workers, children, migrants, immunocompromised patients, and candidates for immunosuppressive therapies. Prior to distribution, the questionnaire was pilot-tested for clarity and relevance prior to distribution and disseminated electronically via professional society mailing lists and social media networks. Responses were anonymous and self-administered.

A total of 101 physicians completed the survey. Their mean age was 42.4 years and 65% were female. Most participants were hospital-based physicians: 84 were identified as hospital practitioners, senior clinical lecturers, or university professors, while 10 were assistants or clinical fellows. Only a few respondents were interns (n = 3) or associate physicians (n = 3). Regarding experience, 34% had been in their current position for 2–5 years, 25% for more than 10 years, and 21% between 5 and 10 years. Notably, 20 respondents had been in their position for less than 2 years. The vast majority of respondents (84%) reported working in departments of infectious diseases, while 6% were specialised in pulmonology, 3% in internal medicine, and 3% in general practice. One respondent reported to work in general hospital medicine. Only 18% of participants reported working within a TB-specific structure such as a TB centre or CLAT (Centre de Lutte Anti-Tuberculeuse), suggesting that the majority operate in general hospital settings without dedicated TB programme affiliation. Regarding diagnostic strategies, a clear preference emerged for interferon-gamma release assays (IGRAs), which were used by 85% of respondents regardless of the manufacturer. This aligns with international recommendations that recognise the superior specificity of IGRAs, particularly in bacille Calmette-Guérin (BCG)-vaccinated populations. In contrast, the tuberculin skin test was reported to be used by only 7% of physicians. This low utilisation rate highlights a progressive disengagement from the method, often perceived as less reliable and more prone to interpretative variability, especially in the context of prior BCG vaccination.

Physicians’ approaches to LTBI management differed markedly depending on the clinical context (Figure). Moreover, a substantial proportion of respondents – including experts – selected ‘I don’t know’, highlighting the complexity and uncertainty that still surround the management of LTBI. Treatment was most consistently recommended in scenarios where the risk of progression to active TB is well recognised. Specifically, 95% of respondents reported offering LTBI treatment to patients scheduled to receive immunosuppressive biotherapies, such as tumour necrosis factor-α inhibitors, and 78% to patients awaiting solid organ transplantation. These results reflect a strong alignment with national and international guidelines that prioritise preventive treatment for immunocompromised individuals due to their elevated risk of reactivation.^6^

**Figure.**
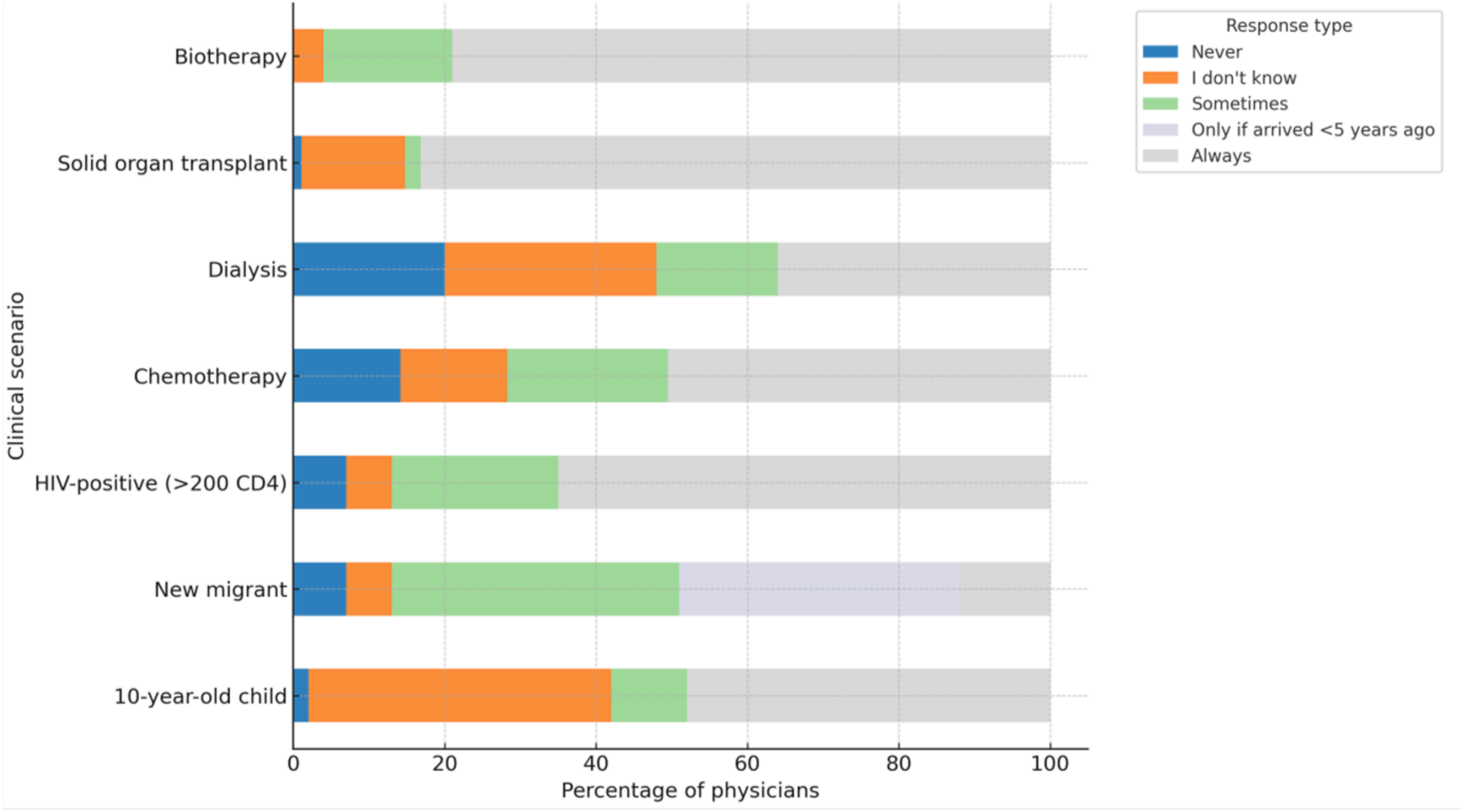
Management of latent TB infection by physicians according to clinical situations (percentage of respondents proposing treatment).

Among HIV-positive individuals, 41.6% of physicians systematically proposed LTBI treatment, while 21.8% did so occasionally. Reported barriers included concerns about drug interactions and patient non-compliance (36.4%). In contrast, management was more heterogeneous in scenarios involving asymptomatic individuals with no overt immunosuppression. Only 8% of respondents systematically proposed LTBI treatment for health care workers identified during routine occupational screening, while 33% reported never offering treatment in this context. Similarly, only 11% of respondents recommended LTBI treatment for migrants regardless of the timing of arrival in France; however, this increased to 29% for the individual who arrived within the last 5 years. For children with LTBI, 41% of respondents reported systematically prescribing treatment. These variations likely reflect both differing interpretations of the evidence-based and physician-perceived benefit–risk ratios in populations where the disease progression risk may be lower or less clearly defined.^6,7^

Treatment practices among respondents were remarkably homogeneous. Most physicians (90%) reported prescribing a combination of rifampicin and isoniazid for a duration of 3 months as their preferred regimen. This short-course regimen is now widely recommended because of its favourable safety profile, higher completion rates, and comparable efficacy to the classic 6–9-month isoniazid preventive therapy. Alternative regimens were infrequently used. A small number of physicians – primarily IDS – reported prescribing isoniazid alone for 6 or 9 months, or rifampicin monotherapy for 4 months. These options were generally reserved for patients with contraindications to one of the agents in the preferred combination or for cases where drug interactions were of specific concern. No statistically significant difference was observed between IDS and pulmonologists regarding preferred regimens (*P* = 0.97), suggesting a strong consensus across specialties. The limited use of multiple regimens may reflect increasing adherence to simplified, evidence-based treatment protocols that favour shorter, well-tolerated regimens usually associated with higher patient’s adherence and reduced adverse events. Acceptance of LTBI treatment varied considerably depending on the clinical scenario. When offered to health care workers as part of routine occupational screening, reported acceptance rates were generally low: only 19% of respondents estimated that treatment was accepted in more than 70% of cases, while approximately one third reported acceptance rates below 50%. These findings suggest persistent reluctance to initiate preventive therapy in this population, likely driven by perceived risks associated with treating a latent condition and concerns about potential adverse effects.^6–8^ In contrast, acceptance rates were reported to be substantially higher in other clinical settings – such as individuals living with HIV, recent migrants, or candidates for immunosuppressive therapies. A majority of respondents estimated that treatment was accepted in over 70% of cases, reflecting greater adherence in contexts perceived to carry a higher risk of progression to active TB.^6,8^

Our data underscore the variability in LTBI management, even among specialists, in a low-incidence country such as France. While practices align with guidelines in high-risk scenarios, they remain inconsistent in others, particularly in asymptomatic populations. These inconsistencies highlight the need for clearer, evidence-based guidance and greater education on the rationale and benefits of LTBI treatment.

## References

[1] World Health Organisation. Global tuberculosis report 2024. Geneva: WHO, 2024.

[2] Sahu S, Overcoming the global tuberculosis crisis with urgent country-level political and financial action. Lancet Infect Dis. 2025;25(1):14-16. 39577455 10.1016/S1473-3099(24)00748-5

[3] HCSP. Infections tuberculeuses latentes. Détection, prise en charge et surveillance Rapport de l’HCSP. Paris, France: Haut Conseil de la Santé Publique, 2019. https://www.hcsp.fr/Explore.cgi/avisrapportsdomaine?clefr=731.

[4] Chaisson RE, Hopewell PC. Rethinking latent TB? Think again. IJTLD Open. 2024;1(8):335-337. 39131593 10.5588/ijtldopen.24.0336 PMC11308401

[5] Dheda K, Migliori GB. New framework to define the spectrum of tuberculosis. Lancet Resp Med. 2024;12(6):426-428. 10.1016/S2213-2600(24)00085-7 38527483

[6] Huaman MA, Sterling TR. Treatment of latent tuberculosis infection-an update. Clin Chest Med. 2019;40(4):839-848. 31731988 10.1016/j.ccm.2019.07.008 PMC7043866

[7] Atchison C, Treating latent TB in primary care: a survey of enablers and barriers among UK general practitioners. BMC Infect Dis. 2015;15:331. 26268227 10.1186/s12879-015-1091-9 PMC4535609

[8] Tang P, Johnston J. Treatment of latent tuberculosis infection. Curr Treat Options Infect Dis. 2017;9(4):371-379. 29238270 10.1007/s40506-017-0135-7 PMC5719124

